# Effect of two phytases at two doses on performance and phytate degradation in broilers during 1–21 days of age

**DOI:** 10.1371/journal.pone.0247420

**Published:** 2021-03-25

**Authors:** Yueming Dersjant-Li, Roger Davin, Trine Christensen, Cees Kwakernaak

**Affiliations:** 1 Danisco Animal Nutrition (IFF), Leiden, The Netherlands; 2 Schothorst Feed Research, Lelystad, The Netherlands; 3 Danisco Animal Nutrition (IFF), Brabrand, Denmark; University of Life Sciences in Lublin, POLAND

## Abstract

The effect of two microbial phytases at two dose-levels on performance and apparent ileal digestibility (AID) of nutrients in broilers fed European-type diets was studied. A total of 1,200 d-old Ross 308 male broilers were randomly assigned to 5 treatments with 30 birds/pen and 8 pens/treatment. A nutritionally adequate positive control (PC) diet was tested against 4 experimental diets containing reduced total P, retainable P, Ca and Na as per the recommended nutritional contribution for *Buttiauxella* phytase (Phy B) at 1,000 FTU/kg (-1.87 g/kg, -1.59 g/kg, -1.99 g/kg and -0.4 g/kg *vs*. PC, respectively). Experimental diets were supplemented with Phy B at 500 FTU/kg or 1,000 FTU/kg, or *Citrobacter* phytase (Phy C) at 1,000 FTU/kg or 2,000 FTU/kg. Diets were based on corn, soybean meal, rapeseed meal and sunflower meal and formulated by phase (starter 1–10 d, grower 11–21 d) in crumbled or pelleted form. Overall (d 1–21), at 1,000 FTU/kg, birds fed Phy C exhibited lower BWG (-2.7%), FI (-3.4%) and tibia ash (-2.2%) vs. PC (*P* < 0.05), and reduced BWG (-3.6%), FI (-3.9%) and tibia ash (-1.8%) vs. Phy B (*P* < 0.05). Phy B at 1,000 FTU/kg and Phy C at 2,000 FTU/kg maintained performance equivalent to the PC. Digestibility of Ca did not differ among phytase treatments but at 1,000 FTU/kg AID P was greater with Phy B than Phy C (72.3% vs. 62.7%, *P* < 0.05). Ileal phytate (myo-inositol hexakisphosphate, IP6) digestibility was greatest with Phy B at 1,000 FTU/kg which was higher than Phy C at 1,000 FTU/kg (87.6 vs. 60.6%, *P* < 0.05). The findings indicate a higher phytate degradation rate of Phy B than Phy C at equivalent dose-level and this is correlated to the performance of the broilers.

## Introduction

The anti-nutritive effects of phytate (the salt form of phytic acid, inositol-6-phosphate, IP6) in commercial broiler diets are well recognized and have been extensively reviewed [1–3]. They arise due to the limited ability of monogastric animals to digest phytate, and its ability to bind with minerals and proteins in the gastrointestinal tract (GIT). This can markedly reduce the bioavailability of not just P, but also other key minerals, amino acids and protein [4], and can impair bird performance and increase excretion of undesirable P into the environment.

Dietary inclusion of exogenous microbial phytase is common practice as a means of increasing the hydrolysis of phytate, improving phytate-P utilization and reducing P excretion [3,5]. ‘Extra-phosphoric’ effects of phytase have also been recognized, for example improvements to the digestibility of amino acids [6–10], sodium absorption in the small intestine [10,11] and energy and starch utilization [8,10,12]. Phytase efficacy is affected by dose; a recent review revealed [3] that traditional dosing at 500 FTU per kilogram of diet typically achieves only 45–60% degradation of phytate (20 to 48% above negative control) by the end of the ileum and that higher dose-levels (1,000 FTU/kg or more) can, but don’t always, increase degradation beyond this range. Olukosi et al. [13] similarly reported increasing phytase dose from 500 to 3000 FTU/kg further increased IP6 degradation rate in broilers fed complex diets containing wheat, barley, soybean meal and rapeseed meal, at 7 (16.5% unit increase) and 28 (31.7% unit increase) days of age. Phytase efficacy is also heavily influenced by dietary Ca content or Ca:P ratio; numerous studies have shown that a high dietary Ca content or Ca:P imbalance can reduce phytase efficacy due to an increased availability of Ca to bind with phytate which limits both Ca and P availability to the animal [14–16]. A recent study showed that fine limestone with highly soluble Ca can have negative impact on phytase efficacy, compared to coarse less soluble limestone [17]. Qian *et al*. [18] suggested an optimal Ca to P ratio within the range of 1.1:1 to 1.4:1 for optimal phytase efficacy. The type of grain (corn versus wheat), and amount of added inorganic P (e.g. mono- or dicalcium phosphate) are also known to impact on the effects of phytase [8,19]. Therefore, when comparing different phytases, it is important to take these factors into account and standardize test conditions as much as possible.

Different generations of commercially available microbial phytases have different pH optima and are therefore likely to be more active/effective in different regions of the gastrointestinal tract [20]. A *Buttiauxella* 6-phytase has recently been shown to be highly active in the proventriculus and gizzard [19] where pH is highly acidic (typically 2.5–3) and where the majority of phytate degradation occurs before it formulates insoluble complexes with minerals such as Ca in the higher pH regions of the small intestine [21]. This phytase may therefore be more effective at degrading phytate than other phytases active at higher pH, especially at higher dose-levels. Recent research showed that 1,000 FTU/kg of *Buttiauxella* phytase was more effective than 500 FTU/kg at improving broiler growth performance (body weight gain: BWG and feed conversion ratio: FCR), particularly in low Ca diets during starter and grower phases [16], but data from direct comparisons with other phytases are limited.

The aim of the current study was to compare directly the effect of a *Buttiauxella* phytase with a *Citrobacter* phytase, on growth performance, phytate and nutrient digestibility in broilers fed typical commercial European type diets containing fine limestone. The phytases were tested at two dose levels, which were different for each phytase but equivalent in terms of their capacity to degrade phytate (IP6) in the ileum when added to corn-soy based diets [22]. The objective was to determine their efficacy at recovering bird performance back to the level produced by a nutritionally adequate diet when added to diets deficient in P and Ca, and to compare their effects on phytate degradation and nutrient digestibility at the two dose-levels.

## Materials and methods

### Birds and housing

The study was carried out according to the guidelines of the Animal and Human Welfare Codes/Laboratory practice codes in the Netherlands and by the Schothorst Feed Research Institute Ethics Review Committee (IvD permit PA17-13) (Schothorst Feed Research, the Netherlands). The treatment, management, housing, husbandry and slaughtering conditions conformed to European Union Guidelines [23].

Ross 308 male broilers were obtained on day of hatch from a local commercial hatchery where they had been vaccinated against infectious bronchitis and assigned to floor-pens on the basis of body weight (BW), so that pens contained birds of approximately equal average bird weight. A total of 1,200 birds were assigned to 40 pens with 30 birds per pen (13.6 chicks/m^2^) and 8 pens per treatment, in a completely randomized block design. Pens contained wood shavings as bedding material and were located in an environmentally controlled broiler house where ambient temperature was maintained initially at 34.5°C and thereafter gradually decreased to 22°C at 21 days of age, under a light-dark (LD) cycle of 23:1 h during the first day and 4D:10L:2D:8L thereafter. Birds were given *ad libitum* access to water and to diets.

### Dietary treatments

The dietary treatments included a positive control (PC) diet and four experimental diets. The PC diet was a corn-soybean meal-based diet formulated to meet the recommended requirements for nutrients of the birds set by the breeder [24]. A fine limestone was used as a common practice in Europe for broiler diets, the particle size was <0.09 mm. The negative control (NC) basal diet was formulated with a reduction of 1.87 g/kg total P, 1.59 g/kg retainable P, 1.99 g/kg Ca and 0.4 g/kg Na, in accordance with the recommended minerals matrix for formulation of diets containing *Buttiauxella* phytase (Phy B) at a dose level of 1,000 FTU/kg feed; reductions were achieved by varying only monocalcium phosphate (MCP), limestone, salt and diamol (filler). The NC was not a stand-alone diet (due to ethical reasons and a NC treatment not being crucial to the objectives of the study), but was supplemented with either Phy B, a *Buttiauxella* sp. phytase expressed in *Trichoderma reesei* or Phy C, a *Citrobacter braakii* phytase expressed in *Aspergillus oryzae*, at one of two dose levels; Phy B was included at 500 or 1,000 FTU/kg and Phy C was included at 1,000 or 2,000 FTU/kg. The reason for selecting different dose-levels across the two phytases was because it has been shown that 500 FTU/kg Phy B is equivalent to a dose level of 1,000 FTU/kg Phy C in terms of the percentage of IP6 degradation in the distal ileum when added to corn-soy based diets [22]. Phytase was dosed based on analyzed product activity by an independent laboratory (LUFA, Nord West, Oldenburg, Germany based on FTU). A common basal diet was produced for each dietary phase, experimental diets were individually mixed by adding the corresponding amounts of monocalcium phosphate, limestone, salt, diamol and the corresponding phytase product and dose-level to the basal diet. Phytase was first premixed in finely ground corn to ensure a homogenous distribution within the feed. One phytase unit (FTU) was defined as the amount of enzyme that released 1 μmol of inorganic orthophosphate from a sodium phytate substrate per minute at pH 5.5 and 37° C [25]. Titanium dioxide (TiO_2_) was added to all diets at a level of 3.5 g/kg as the inert marker. All diets were pelleted (3 mm pellet diameter) with a target pellet temperature of ≤80°C, and starter diets were subsequently crumbled. The composition of the PC and NC basal diets is given in Table 1.

**Table 1:** Ingredient and chemical composition (g/kg, as fed basis) of the positive control and basal (NC) diets.

	Starter (d 1–10)		Grower (d 11–21)	
	PC	NC^a^	PC	NC^a^
***Ingredient*, *g/kg***				
Corn	558.8	558.8	567.9	567.9
Soybean meal, 48% CP	331.9	331.9	267.0	267.0
Rapeseed meal	38.0	38.0	40.0	40.0
Sunflower seed meal	0.0	0.0	40.0	40.0
Lard	20.1	20.1	35.3	35.3
Soybean oil	4.7	4.7	4.7	4.7
Lysine HCl, 79%	2.3	2.3	2.6	2.6
DL-Methionine, 99%	2.7	2.7	2.4	2.4
L-Threonine, 98%	0.9	0.9	0.8	0.8
Sodium bicarbonate	1.5	1.5	1.5	1.5
Vitamin+mineral premix^b^	5.0	5.0	5.0	5.0
Coccidiostat^c^	1.0	1.0	1.0	1.0
Titanium dioxide	-	-	3.5	3.5
Monocalcium phosphate	14.2	5.9	11.8	3.5
Limestone, < 0.09 mm	15.6	14.1	13.2	11.7
Salt	3.3	2.3	3.3	2.3
Diamol	0.0	10.8	0.0	10.8
***Calculated nutrients*, *g/kg***				
AME, kcal/kg	2,850	2,850	2,925	2,925
Crude Protein	221.0	221.0	205.0	205.0
Total Lysine	13.6	13.6	12.5	12.5
Digestible Lysine	12.0	12.0	11.0	11.0
Total Methionine + Cystine	9.8	9.8	9.1	9.1
Digestible Methionine + Cystine	8.60	8.61	8.02	8.03
Total phosphorus (tP)	7.2	5.3	6.7	4.8
Phytate phosphorus (PP)	2.5	2.5	2.7	2.7
Available P (Av P)	4.6	2.7	4.0	2.2
Retainable P	4.2	2.6	3.7	2.1
Calcium, Ca	9.6	7.6	8.4	6.4
Sodium, Na	1.8	1.4	1.8	1.4
Chlorine, Cl	3.1	2.5	3.2	2.5
Potassium, K	9.8	9.8	9.0	9.0
Dietary electrolyte balance, dEB, meq/kg	244	244	221	221
***Analyzed nutrient composition*, *g/kg***				
Crude Protein	214.0	214.0	206.0	206.0
Total P	7.0	5.3	6.6	4.8
Phytate P	2.7	2.7	2.9	2.9
Av P (calculated)	4.3	2.6	3.7	1.9
Ca	9.7	8.2	8.0	6.5
Na	1.8	1.4	1.7	1.4

^a^ NC: Negative control basal diets were not stand-alone diets but supplemented with phytase according to treatment. Phytase was dosed based on analyzed product activity by an independent laboratory (LUFA, Nord West, Oldenburg, Germany).

^b^ Supplied per kg of diet: Vitamin A, 10,000 IU; vitamin D3, 2,400 IU; vitamin E, 50 mg; vitamin K3, 1.5 mg; vitamin B1, 2.0 mg; vitamin B2, 7.5 mg; pantothenic acid, 12 mg; niacin, 35 mg; biotin, 200 μg; vitamin B12, 20 μg; folic acid, 1.0 mg; vitamin B6, 3.5 mg; choline chloride 460 mg; Fe, 80 mg (as FeSO_4_.H_2_O); Cu, 12 mg (as CuSO_4_.5H_2_O); Zn, 60 mg (as ZnSO_4_.H_2_O); Mn, 85 mg (as MnO); I, 0.8 mg (as KI); Se, 0.15 (as Na_2_SeO_3_); Co, 0.4 mg (as CoSO_4_.7H_2_O).

^c^ Clinacox^®^ in starter and Sacox^®^ in grower diets.

### Sampling and measurements

Body weight and feed intake (FI) were measured per pen on d 1, 10 and 21 and used to calculate average BW gain (BWG) and mortality corrected FCR. Mortality was recorded daily.

On d 21, 12 birds per pen were euthanized by CO_2_ asphyxiation and the left tibias of 4 birds obtained and pooled for determination of de-fatted tibia ash content. Tibias were stripped of adjacent tissues and dried over-night initially at 40°C and subsequently overnight at 70°C. Fat was extracted from the tibias using a Soxhlet apparatus and 100% petroleum ether according to modified methods of Watson et al. [26]. Fat-extracted-tibias were dried for 4 h at 103°C and ashed in a muffle furnace for 24 h at 700°C to determine bone ash content.

Digesta was obtained from all 12 euthanized birds per pen, by gentle squeezing from the last 25 cm of the ileum as described by [27]. Ileal digesta samples were pooled, freeze-dried and ground to pass through a 1 mm sieve prior to nutrient analysis. Ileal digestibility of nutrients was calculated using TiO_2_ as an indigestible marker. Representative sub-samples of all dietary treatments were analyzed for Ca, P and phytate content.

### Chemical analysis

Samples were analyzed in duplicate for all analyses. The dry matter content of diets and digesta was determined by drying overnight in a 103°C force-draft oven, according to the ISO 6496 [28] procedure. Crude protein in diets was analyzed according to ISO 16634–2 [29]. Dietary and ileal Ca were determined after acid digestion and analyzed using inductively coupled plasma atomic emission spectrometry (ICP-AES) [30]. Titanium (Ti) and P in diets and digesta were determined by calorimetry according to a method adapted from Short *et al*. [31] and AOAC method 986.24 [32]. Sodium was analyzed by the AAS (dry ashing) method (NEN/ISO 6869) [33]. Phytate phosphorus (PP [inositol hexa-phosphate (IP6)]) concentrations in diets and digesta (blind samples for treatment details and labeled by pen number) were determined by DuPont Innovation Laboratories (Brabrand, Denmark) using a modified version of the HPLC method described by Skoglund et al. [34]. In short, detection of IP6 was obtained using an anion exchange column (4 × 250 mm with a 4 × 50 mm pre-column; CarboPac Guard PA-100; Dionex, Sunnyvale, CA). MilliQ water and 0.5 N HCl were used as Solvents A and B, respectively and the isocratic program used 100% (vol/vol) Solvent B for a duration of 10 min. The flow rate was 1.0 mL/ min. IP6 standards (Sigma-Aldrich) was prepared in eluent B and analyzed under the same conditions. The eluant from the PA-100 column was reacted in-line in a T-tube with Fe^3+^ perchloric acid solution at a flow rate 0.4 mL/min IP6 were detected at 290 nm as positive peaks resulting from the formation of IP6-Fe^3+^-ClO_4_− complex. The linear range for IP6 was 10 to 225 mg/L with an injection volume of 25 μL and IP6 concentration of samples was determined accordingly. Extraction of IP6 from the feed and digesta was carried out using 0.5M HCl as solvent and at a sample concentration of 0.05 g/mL. For extraction, samples were rotated on a mixer for 1 h at room temperature and subsequently centrifuged. Supernatants were filtered (0.45 μM) and subjected to analysis. For digesta samples, extraction procedure was repeated of pellets at a sample concentration of 0.1 g/mL before subjecting to analysis by HPLC. For both diet and digesta samples the IP6 content was quantitated against the standard curve respectively as a percentage (g IP6 per 100 g of feed) and % IP6 in digesta DM (based on total IP6 from both extractions). Phytase activities in the diets were determined by Eurofins (Denmark) using the ISO/DIS 30024 [35] procedure.

### Calculations

Apparent ileal digestibility (AID; %) of P, Ca and phytate (IP6) were calculated based on the following formula, using TiO_2_ as the inert marker:

$$AID=(1–(Ti_{d}/Ti_{i}\times N_{i}/N_{d}))*100$$

Where Ti_d_ is the titanium concentration in the diet, Ti_i_ is the titanium concentration in the ileal digesta, N_i_ is the nutrient (P, Ca or phytate (IP6)) concentration in the ileal digesta and N_d_ is the nutrient concentration in the diet. All analyzed values were expressed as grams per kilogram dry matter.

### Statistical analyses

Data were analyzed on a per pen basis, by one-way analysis of variance (ANOVA) using the Fit Model platform of JMP 14.0 (SAS Institute Inc., Cary, NC, 1989–2019). Treatment was considered as a fixed effect. Means separation was achieved using Tukey’s Honest Standard Difference test. Differences were considered significant at *P* < 0.05. *P* < 0.10 was considered a trend.

## Results

### Enzyme recoveries and analyzed nutrients

Analyzed values of P, phytate-phosphorus (PP), Ca and Na concentration in the control and experimental diets were all within 10% of calculated values (Tables 1 and 2). Analyzed phytase activities were less than 50 FTU/kg in the PC diets, suggesting no cross-contamination among control and phytase-supplemented diets, and were within 20% of target values in all experimental diets except the grower phase diets for the 500 FTU/kg Phy B and 2,000 FTU/kg PhyC treatments, where analyzed values were 27% higher than target values (Table 2).

**Table 2:** Analyzed phytase activity^a^ and nutritional values (g/kg) in the positive control (PC) and experimental diets (NC plus phytase).

	PC	Phy B^b^ FTU/kg		Phy C^b^ FTU/kg	
		500	1,000	1000	2,000
**Starter, d 1–10**					
Phytase activity, FTU/kg	<50	527	893	1,134	2,387
Ca, g/kg	9.66	7.86	8.20	8.03	8.31
P, g/kg	6.95	5.26	5.24	5.29	5.18
**Grower, d 11–21**					
Phytase activity, FTU/kg	<50	633	928	815	2,537
Ca, g/kg	8.01	6.60	6.41	6.35	6.45
P, g/kg	6.49	4.80	4.77	4.76	4.79

^a^Phytase activity in feed was analyzed by Eurofins (Denmark).

^b^Phy B: A *Buttiauxella* phytase expressed in *Trichoderma reesei;* Phy C: A *Citrobacter* phytase expressed in *Aspergillus oryzae*.

### Growth performance and tibia ash

Effects of the two phytases on growth performance are presented in Table 3. During the starter phase (d 1–10), all phytase treatments maintained performance parameters (BWG, FI and FCR) to the level of the PC, except Phy C at 1,000 FTU, which had reduced feed intake vs PC. However, Phy B at a dose of 1,000 FTU/kg produced a greater (*P* < 0.05) BWG than Phy C at the same dose (+4.2%) or than Phy B at 500 FTU/kg (+4.0%). Phy C at the lower dose-level (1,000 FTU/kg) resulted in a lower FI than 1,000 FTU/kg Phy B (*P* < 0.05) and a lower FCR than 500 FTU/kg (but not 1,000 FTU/kg) Phy B (*P* < 0.05). There were no differences in mortality across treatments during the starter phase.

**Table 3:** Effect of two phytases (Phy B and Phy C) at two doses on growth performance (1–21 d) and tibia ash at 21 d in broilers.

	PC	Phy B^a^ FTU/kg		Phy C^a^ FTU/kg		SEM	*P*-value
		500	1,000	1,000	2,000		
Starter, d 1–10							
**BWG, g/bird**	258.3^ab^	250.3^b^	260.2^a^	249.8^b^	254.0^ab^	2.328	0.006
**FI, g/bird**	278.1^a^	273.4^ab^	281.4^a^	268.5^b^	274.3^ab^	2.24	0.003
**FCR, g/g**	1.077^ab^	1.093^a^	1.081^ab^	1.075^b^	1.080^ab^	0.004	0.042
**Mortality, %**	1.95	0.39	0.39	0.78	0.0	0.622	0.225
Grower, d 11–21							
**BWG, g/bird**	821.4^ab^	804.0^b^	828.6^a^	802.0^b^	816.4^ab^	5.833	0.006
**FI, g/bird**	1043.2^ab^	1017.8^bc^	1046.0^a^	1009.3^c^	1035.4^abc^	7.06	0.001
**FCR**	1.270	1.266	1.263	1.258	1.268	0.004	0.345
**Mortality, %**	1.56	1.16	0.00	0.39	0.78	0.535	0.279
Overall, d 1–21							
**BWG, g/bird**	1080.8^ab^	1053.8^bc^	1091.3^a^	1051.6^c^	1070.4^abc^	6.643	0.0005
**FI, g/bird**	1323.1^a^	1288.6^b^	1329.6^a^	1277.6^b^	1309.6^ab^	8.670	0.0003
**FCR**	1.224	1.223	1.218	1.215	1.224	0.003	0.119
**Mortality, %**	3.51^a^	1.56^ab^	0.39^b^	1.16^ab^	0.78^ab^	0.817	0.065
**Tibia ash, g/kg DM**	510^a^	502^bc^	508^ab^	499^c^	506^abc^	3.153	0.004

^a^Phy B: A *Buttiauxella* phytase expressed in *Trichoderma reesei;* Phy C: A *Citrobacter* phytase expressed in *Aspergillus oryzae*.

^a,b,c^ Means in the same row with no common superscripts are significantly different (*P* < 0.05).

During the grower phase (d 11–21), effects on BWG were similar to that seen in the starter phase. All phytase treatments maintained BWG to the level of the PC, and BWG was again greater in birds supplemented with Phy B at 1,000 FTU/kg than in birds supplemented with Phy C at the same dose or with Phy B at 500 FTU/kg (+3.3% and +3.1%, respectively) (*P* < 0.05; Table 3). Feed intakes of all phytase treatment groups were similar to the PC, except for the Phy C 1,000 FTU/kg group which exhibited a reduced feed intake (-3.2% *vs*. PC; *P* < 0.05). In addition, FI was higher in birds supplemented with 1,000 FTU/kg Phy B compared with birds supplemented with 500 FTU/kg Phy B or 1,000 FTU/kg Phy C (*P* < 0.05). There were no differences in FCR and mortality across treatment groups during grower phase.

For the overall period (d 1–21), birds supplemented with 1,000 FTU/kg Phy C exhibited reduced BWG compared with 1,000 FTU/kg Phy B (-3.6%) or the PC (-2.7%; *P* < 0.05), whilst Phy B at both dose-levels and Phy C at 2,000 FTU/kg maintained BWG compared with the PC. Feed intakes were lower than PC in both the Phy B 500 FTU/kg group and the Phy C 1,000 FTU/kg groups (*P* < 0.05) but were equivalent to PC in the Phy B 1,000 FTU/kg and Phy C 2,000 FTU/kg treatments. No differences in FCR were evident across treatments for the overall phase. Mortality tended to be lower in the Phy B 1,000 FTU/kg compared with the PC (0.39% vs. 3.51%, respectively, *P* = 0.065).

Only Phy B at 1,000 FTU/kg and Phy C at 2,000 FTU/kg maintained tibia ash at a level equivalent to PC, while tibia ash was reduced in birds fed the lower dose levels of either enzyme (500 FTU/kg of Phy B (*P* < 0.05) or 1,000 FTU/kg of Phy C (*P* < 0.05)).

### Nutrient digestibility

Apparent ileal digestibility of phytate (IP6) was, as expected, markedly improved by phytase supplementation (*P* < 0.001; Table 4). The greatest effect was seen with Phy B at 1,000 FTU/kg, which achieved an extra 27 percentage points in phytate degradation compared with the same dose of Phy C (87.6 *vs*. 60.6%. *P* < 0.05). Ileal digestibility of P and Ca were also improved by all phytase treatments *vs*. PC (*P* < 0.05); P digestibility was greatest with Phy B at 1,000 FTU/kg (AID P 72.3%) and greater compared to Phy C at the same dose-level (AID P 62.7%, *P* < 0.05). High phytase dose further increased ileal digestibility of IP6 and P, however, Phy B at 500 FTU was equal to Phy C at 1,000 FTU, and Phy B at 1,000 FTU was equal to Phy C at 2,000 FTU. Ileal digestibility of Ca was improved equally by all phytase treatments versus PC (*P* < 0.05).

**Table 4:** Effect of two phytases (Phy B and Phy C) at two doses on apparent ileal digestibility (AID) of P, Ca, phytate (IP6) in ileal digesta in broilers at 21 days of age.

	PC	Phy B^a^ FTU/kg		Phy C^a^ FTU/kg		SEM	*P*-value
		500	1000	1000	2000		
**AID P, %**	48.3^c^	61.3^b^	72.3^a^	62.7^b^	71.0^a^	1.108	< 0.001
**AID Ca, %**	37.7^b^	52.7^a^	54.6^a^	50.7^a^	54.9^a^	1.283	< 0.001
**AID phytate IP6), %**	-1.0^c^	68.3^b^	87.6^a^	60.6^b^	79.2^a^	2.540	< 0.001

^a^Phy B: A *Buttiauxella* phytase expressed in *Trichoderma reesei;* Phy C: A *Citrobacter* phytase expressed in *Aspergillus oryzae*.

^a,b,c,d^ Means in the same row with no common superscripts are significantly different (*P* < 0.05).

## Discussion

Analyzed phytase in the experimental treatments was broadly consistent with target dose-levels. In the two treatments where analyzed activities were higher-than-target (+27%), this may be due to the diet mixing and sampling. Thus, we consider that observed effects of treatment could reasonably be ascribed to the assigned dose-levels. Based on analyzed values, P and Ca in the PC diets matched well with formulated values (within 10%), as did the reductions in P and Ca achieved in the experimental diets. The dietary ratios of Ca to P (total, analyzed) in the PC diets were 1.39:1 and 1.15:1 respectively for starter and grower phases, within the range proposed by Qian *et al*. [18] for optimal phytase efficacy (1.1:1 to 1.4:1).

The positive effect of exogenous microbial phytase on phytate degradation and P digestibility in broilers is well established and has been extensively discussed [3,5,19]. Data from the present study add to the existing evidence. Both phytases at both dose-levels were effective at hydrolyzing phytate and releasing phytate-bound P, so that by the end of the ileum, all treatments had improved the AID of phytate compared to PC. The values obtained for AID of phytate and P with *Buttiauxella* phytase at 1,000 FTU/kg (87.6% and 72.3%, respectively) are consistent with recent studies by Li *et al*. [36], Kim *et al*. [17] and Dersjant-Li *et al*. [16] in comparable diets with the same phytase and at the same dose-level, suggesting a consistency of effect across multiple settings.

However, the two phytases differed markedly in their capacity to degrade phytate and release phytate bound-P under the tested conditions, and this was not solely related to dose-level. This was evidenced by a greater improvement in phytate (IP6) digestibility (+27 percentage points) and (total) P digestibility (+9.6 percentage points) with *Buttiauxella* phytase dosed at 1,000 FTU/kg than with *Citrobacter* phytase dosed at the same level. At 500 FTU/kg, the *Buttiauxella* phytase produced equivalent phytate degradation and ileal P digestibility to 1,000 FTU/kg of the *Citrobacter* phytase. This suggests that per standard FTU the *Buttiauxella* phytase was more efficacious in the tested setting. In fact, Phy B at 1,000 FTU had numerically greater ileal digestibility of IP6 than 2,000 FTU/kg of Phy C (+8.4 percentage points). This indicates greater relative efficacy of Phy B across a broad commercial dose range.

The different efficacy of the phytases per standard FTU could be related to their particular enzymatic properties and proposed mode of action in the GIT. Phytase is dosed in animal feed based on a standardized activity at pH 5.5, but relative activity at lower pH such as that encountered in the gizzard (where pH is around 3.0 or below), can differ markedly among phytases. For example, the *Buttiauxella* phytase has a relative activity of 235% at pH 3.0 (relative to pH 5.5) [20], and is more active at this low pH than other phytases such as *Citrobacter* phytase which exhibits a relative activity of 146% at pH 3.0 [20]. Therefore *in vivo*, the *Buttiauxella* phytase is predicted to be more active than *Citrobacter* phytase in the low pH of the upper GIT. In fact, Lee et al. [37] observed that pH in the gizzard can be much lower than 3.0, reporting capsule readings from pH 0.54 to 4.84 across different dietary treatments. Several previous studies have indicated a high activity of *Buttiauxella* phytase in the proventriculus and gizzard for degrading phytate (IP6) [22,37,38]. In addition, a recent comparative study reported a greater dose-equivalent effect of *Buttiauxella* phytase compared with an *E*. *coli* phytase on ileal P digestibility, retention and growth performance in broilers fed diets comparable to the present study [11], like *Citrobacter* phytase, *E*. *coli* phytase has also been shown to be less active than Buttiauxella phytase at pH 3.0 [20].

A higher efficacy of the *Buttiauxella* phytase in increasing P availability may also have resulted from it effecting a more complete breakdown of IP6 in the upper GIT digesta. Li *et al*. [38] reported an ileal IP6 digestibility of up to 91% in broilers fed diets supplemented with *Buttiauxella* phytase at 1,000 FTU/kg. Bello *et al*. [22] observed a significantly greater ileal IP6 digestibility with *Buttiauxella* phytase compared with the same dose of *Citrobacter* phytase. The IP6 digestibility values reported by Bello *et al*. [22] for *Buttiauxella* phytase compare well with those reported in the present study. The negative AID phytate P value observed in the present study may have been due to (acceptable) analytical error in the phytate-P analysis caused by incomplete extraction of phytate. Nevertheless, it indicates that with a diet containing highly soluble limestone (Ca solubility was 100% within 10 min at pH 3.0), the phytate degradation rate in the control diets was low.

Calcium digestibility was improved uniformly by all phytase treatments compared with the PC, independent of phytase source or dose-level. This may have arisen if Ca requirements of the birds were already met with the lowest dose-levels of either phytase. However, Dersjant-Li and Kwakernaak [11] observed that increasing Phy B from 250 to 1,000 FTU/kg did not increase ileal Ca absorption but improved total tract Ca retention, which suggests that total tract Ca retention may be a more sensitive parameter than Ca digestibility for evaluating effects of phytase on Ca utilization, when diets were excess in Ca relative to available P.

The observed improvements in ileal phytate degradation, P and Ca digestibility in all phytase treatments were sufficient to recover growth performance back to a nutritionally complete PC diet, except that *Citrobacter* phytase at 1,000 FTU/kg and *Buttiauxella* phytase at 500 FTU/kg reduced feed intake. In all phytase treatments during either phase, FCR values were generally within 10% of performance objectives for the breed (Aviagen; http://www.en.aviagen.com (accessed 18 October 2018)), but there were some differences in BWG and FI between treatments that appeared to be related to dose-level and phytase source (*Buttiauxella* or *Citrobacter*). In addition, for the overall phase (d 1–21), *Buttiauxella* phytase achieved similar overall performance (BWG and FCR) to the PC at either dose-level, but *Citrobacter* phytase only achieved equivalent performance to the PC at the higher dose (2,000 FTU/kg). These findings support the digestibility data in suggesting a greater dose-equivalent effect of *Buttiauxella* phytase than *Citrobacter* phytase at 1,000 FTU/kg. At this dose-level, *Buttiauxella* phytase produced birds with greater overall BWG, FI and tibia ash than birds supplemented with *Citrobacter* phytase. Although FCR was lower in birds supplemented with *Citrobacter*- at 1,000 FTU compared to *Buttiauxella* phytase at 500 FTU during the starter phase (d 1–10), this likely resulted from both BWG and FI having been reduced in the latter group, rather than reflecting a meaningful improvement in BWG in relation to FI per se. *Buttiauxella* phytase at 500 FTU/kg produced a similar phytate degradation rate compared to *Citrobacter* phytase at 1,000 FTU/kg, but the latter did not recover 1-21d BWG and feed intake back to the level of the PC. This may be an indication that the *Buttiauxella* phytase produced a greater extra-phosphoric effect than *Citrobacter* phytase. Interestingly, *Buttiauxella* phytase at the higher dose-level was also associated with a reduced level of mortality compared with the PC (0.39% vs. 3.51%). It is unclear whether this was coincidental or causal. In this study, a true NC with P and Ca deficient diet was not included due to the animal welfare reason and because a NC alone treatment is not critical for this study. However, at low phytase dose, the tibia ash was significantly lower than PC, indicating low phytase dose was not able to compensate 1.87 g/kg total P, 1.59 g/kg retainable P, 1.99 g/kg Ca reduction to maintain the tibia ash. High dose of phytase (*Buttiauxella* phytase at 1,000 FTU/kg and *Citrobacter* phytase at 2,000 FTU/kg) was needed to recover the tibia ash to the level of PC.

Finally, there also appeared to be an effect of bird age on the effects of phytase. The degree of improvement in performance (BWG) with *Buttiauxella* phytase at 1000 FTU/kg *vs*. *Citrobacter* phytase at the same dose (1,000 FTU/kg) was greater in younger birds (d 1–10) than older birds (d 11–21). Furthermore, the effect of increasing dose of *Buttiauxella* phytase from 500 FTU/kg to 1,000 FTU/kg on BWG was also greater in younger than in older birds. A comparable finding was recently reported by Li *et al*. [39] in relation to AID of P and Ca; the authors observed that the beneficial effects of increasing *Buttiauxella* phytase dose from 500 FTU/kg to 1,000 FTU/kg were greater in younger (d 9) than older birds (d 21). As discussed by Li *et al*. [39], it seems likely a faster transit time and less well developed digestive tract of younger birds may contribute to the greater apparent capacity for younger birds to benefit from phytase supplementation at 1,000 to 2,000 FTU/kg.

## Conclusion

This study has demonstrated that under the tested conditions the degree of efficacy differed between the phytase source and dose-level applied. Overall (1–21 d), *Buttiauxella* phytase, added to diets with reduced P and Ca at both 500 and 1,000 FTU/kg maintained BWG and at 1000 FTU/kg maintained tibia ash compared to a nutritionally adequate PC, but a *Citrobacter* phytase was able to maintain these performance measures only at a dose-level of 2,000 FTU, not 1,000 FTU/kg. At an equivalent dose-level of 1,000 FTU/kg, *Buttiauxella* phytase produced greater BWG, a higher ileal digestibility of phytate (IP6), total P and tibia ash than *Citrobacter* phytase, which is hypothesized to have been related to a higher capacity to degrade IP6 in the upper GIT where *Buttiauxella* phytase is most active.
